# Preparation and Evaluation of *Undaria pinnatifida* Nanocellulose in Fabricating Pickering Emulsions for Protection of Astaxanthin

**DOI:** 10.3390/foods11060876

**Published:** 2022-03-18

**Authors:** Yu Li, Siyuan Fei, Deyang Yu, Lijuan Zhang, Jiaxuan Li, Ronggang Liu, Mingqian Tan

**Affiliations:** 1 Academy of Food Interdisciplinary Science, School of Food Science and Technology, Dalian Polytechnic University, Qinggongyuan 1, Dalian 116034, China; liyu06190908@163.com (Y.L.); 13470319363@163.com (S.F.); yudy0411@163.com (D.Y.); zhanglijuan202202@163.com (L.Z.); nancylee98@126.com (J.L.); lrg19950427@163.com (R.L.); 2 National Engineering Research Center of Seafood, Dalian Polytechnic University, Dalian 116034, China; 3 Collaborative Innovation Center of Seafood Deep Processing, Dalian Polytechnic University, Dalian 116034, China

**Keywords:** *Undaria pinnatifida*, nanocellulose, Pickering emulsion, stability, astaxanthin

## Abstract

Pickering emulsions stabilized from natural sources are often used to load unstable bio-active ingredients, such as astaxanthin (AXT), to improve their functionality. In this study, AXT-loaded Pickering emulsions were successfully prepared by 2,2,6,6-tetramethy-1-piperidine oxide (TEMPO)-oxidized cellulose nanofibers (TOCNFs) from *Undaria pinnatifida*. The morphology analysis showed that TOCNFs had a high aspect ratio and dispersibility, which could effectively prevent the aggregation of oil droplets. The stable emulsion was obtained after exploring the influence of different factors (ultrasonic intensity, TOCNFs concentration, pH, and ionic strength). As expected, AXT-loaded Pickering emulsions showed good stability at 50 °C and 14 days of storage. The results of simulated *in vitro* digestion showed that the emulsions exhibited higher release of free fatty acids (FFAs) and bioaccessibility of AXT than those in sunflower oil. Hence, our work brought new insights into the preparation of Pickering emulsions and their applications in protection and sustained, controlled release of AXT.

## 1. Introduction

In recent years, more and more attention has been paid to the development of health food containing natural active ingredients. Astaxanthin (AXT), a natural carotenoid produced from salmon, crab, shrimp, and algae [1], has strong antioxidant properties, which has been widely applied in functional foods. However, due to the structure of unsaturated double bonds, AXT is easily decomposed under the conditions of oxygen, light, and heat. In addition, AXT has relatively poor water solubility and dispersion, leading to serious loss of its nutritional value [2]. Moreover, mammals cannot synthesize AXT in their own body and must take AXT from food items [3]. Therefore, it is of vital importance to build a delivery system, such as an emulsion, to encapsulate the hydrophobic natural active ingredients such as AXT for effective oral administration [4].

Compared with traditional emulsions, Pickering emulsions have better stability and can improve the nutrition, function and bioaccessibility of bioactive substances [5]. At present, the study of Pickering emulsions holds great promise by using green and degradable natural solids instead of synthetic small-molecule surfactants [6]. Nanocellulose is one kind of environmentally friendly nanomaterials that can be extracted from natural, renewable plant-based sources [7] and has gained wide attention as a stabilizer of Pickering emulsions due to its high specific surface area and uniform particle-size distribution [8]. Being a typical type of nanocellulose, cellulose nanofibers (CNFs), ranging from tens of nanometers to several microns in length, exhibit unique characteristics, such as high specific surface area, good surface chemical activity, and low thermal expansion coefficient [9]. Generally, the CNFs could be obtained through mechanical processes, such as high-pressure homogenization, ball milling, and high-speed shearing [10]. However, these mechanical processes require a large amount of energy and need a pretreatment to avoid a large area of clogging [11]. Therefore, it is highly desirable to develop an environmentally friendly, low-cost, and convenient strategy to obtain CNFs, such as cellulase hydrolysis. After enzymatic hydrolysis, the cellulose molecular chain still has a large number of hydrogen bonds between hydroxyl groups and oxygen atoms, making nanocellulose insoluble in general solvents and reducing its application [12]. To this end, 2,2,6,6-tetramethy-1-piperidine oxide (TEMPO)-mediated oxidation has been used as an effective method that can regioselectivity oxidize the C6-primary hydroxyl groups to C6-carboxylate with little damage [13]. The TEMPO-oxidized cellulose nanofibers (TOCNFs) are more stable at the oil–water interface, protecting droplets in the emulsion from polymerization. Therefore, TOCNFs have great potential as an emulsifier to prepare Pickering emulsions and stabilizing fats for different kinds of foods [14,15]. Moreover, ultrasound-assisted chemical treatment can induce the cavitation effect, which is able to effectively separate TOCNFs without destroying its structure, and obtain different size ranges of TOCNFs [16].

CNFs are often obtained from terrestrial woody plants. In fact, marine algae have great potential as a source for CNFs isolation [17]. *Undaria pinnatifida*, one of the typical brown algae, show great advantages in the preparation of nanocellulose. It does not require land to plant, thus reducing the utilization of land resources. Moreover, the cell walls of algae contain almost no lignin, making the extraction and purification of cellulose more efficient [18]. However, there is hardly any work reporting the preparation and application of cellulose nanofibers from *Undaria pinnatifida* in fabricating Pickering emulsion for AXT encapsulation.

In this study, a combination of enzymatic hydrolysis and TEMPO oxidation was developed to prepare TOCNFs from *Undaria pinnatifida*. Subsequently, the TOCNFs were thoroughly characterized and used to prepare a Pickering emulsion. By investigating the effects of ultrasonic power, TOCNFs concentration, ionic strength, and pH on the emulsion, the Pickering emulsion was prepared. Furthermore, AXT-loaded Pickering emulsion stabilized with TOCNFs and its thermal stability and storage stability were also evaluated. Finally, the release of free fatty acids (FFAs) of AXT-loaded Pickering emulsion and the bioaccessibility of AXT were examined by *in vitro* simulated digestion.

## 2. Materials and Methods

### 2.1. Materials

*Undaria pinnatifida* was purchased from the local market in Dalian, China. NaOH, Na_2_CO_3_, NaClO, and CH_3_COOH were purchased from Aladdin Chemical Reagent Co., LTD (Shanghai, China). Na_3_PO_4_·12H_2_O, Na_2_SiO_3_·9H_2_O, TEMPO, NaBr, NaCl, HCl, and CaCl_2_ were purchased from Macklin Biochemical Technology Co., LTD (Shanghai, China). All the reagents were of analytical grade. AXT crude (10% purity) was purchased from Xi’an Realin Biotechnology Co., LTD (Xi’an, China). Cellulase (5 × 10^4^ U/g) was purchased from Jiangsu Ruiyang Biotechnology Co., LTD (Jiangsu, China). Pepsin (porcine gastric mucosa, ≥500 U/mg) and standard AXT (purity > 95%) were purchased from Sigma-Aldrich Co., LTD (St Louis, MO, USA). Trypsin (130 U/mg), and lipase (1 × 10^5^ U/g) was purchased from Macklin Biochemical Technology Co., LTD (Shanghai, China). Bile salt was purchased from Qingdao Hopebio Biotechnology Co., LTD (Qingdao, China). Deionized water was used for all the experiments.

### 2.2. Preparation of TOCNFs

#### 2.2.1. Preparation of *Undaria pinnatifida* Cellulose (U-Cellulose) by Alkali and Bleaching Treatments

The fresh *Undaria pinnatifida* was washed repeatedly with water to remove solid surface impurities. Subsequently, the clean *Undaria pinnatifida* was dried in an oven at 60 °C for two days. After drying, it was ground into powder and passed through a mesh with pore size of 0.15 mm. U-cellulose was obtained according to the previous methods [19,20]. Initially, 20 g of dried *Undaria pinnatifida* powder were added to an aqueous solution containing 1 wt% Na_2_CO_3_ in a ratio of 1:50 (*w*/*v*) and stirred at 105 °C for 2 h. After centrifugation at 4 °C with the speed of 8000 rpm for 10 min, the precipitate was washed repeatedly for three times with deionized water and redispersed into an aqueous solution containing 2 wt% NaOH at constant volume to 1 L. The precipitate was obtained by centrifugation after reaction at 120 °C for 2 h. The NaOH treatment process was repeated 3 times to remove the additional alginate and other impurities. After alkali treatment, 1 L of sodium acetate buffer solution (3.5 wt% NaOH and 75 CH_3_COOH) and 20 mL of NaClO were added to the final reactants at 80 °C for 2 h. A white precipitate was obtained by centrifugation. To avoid hemicellulose or lignin in the obtained cellulose, the precipitate was added in a 1 L aqueous solution containing 2 wt% NaOH, 1 wt% Na_3_PO_4_·12H_2_O, and 1 wt% Na_2_SiO_3_·9H_2_O and was treated at 100 °C for 1.5 h. After that, the precipitate was repeatedly washed, centrifuged, and then dialyzed using a dialysis membrane with an intercepted molecular weight of 3500 kDa. Finally, 11.2 g of U-cellulose was obtained after being freeze-dried.

#### 2.2.2. Preparation of CNFs by Enzymatic Hydrolysis

CNFs were prepared by enzymatic hydrolysis method as reported by Wu et al. [21] with slight modifications. In brief, 1 g of freeze-dried U-cellulose was added to 500 mL of deionized water, followed by adding 100 mg of cellulase (0.2 mg/mL). Then, the pH value of the suspensions was adjusted to 4.8 using 0.1 mol/L NaOH and 0.1 mol/L HCl. The hydrolysis reaction was conducted at 60 °C for 3 h. After reaction, the CNFs were centrifuged twice at 10,000 rpm for 15 min to remove excess cellulase, and 0.54 g of CNFs were obtained after being freeze-dried.

#### 2.2.3. Preparation of TOCNFs

The preparation of TOCNFs was carried out according to the method of Zhou et al. [22] with slightly modifications. The CNFs were oxidized by the TEMPO/NaBr/NaClO by adding 1 g of CNFs in 200 mL aqueous solution containing TEMPO (0.1 mmol/g cellulose) and NaBr (1 mmol/g cellulose). Then, 7.14 mL of NaClO solution (6–14% active chlorine basis, 10 mmol/g cellulose) was added dropwise into the CNFs suspension at room temperature under magnetic stirring. The pH of the suspension was adjusted to pH 10 ± 0.2 during the reaction. When the pH of the suspension did not change for about 10 min, the oxidation reaction was considered to be completed. The oxidation reaction was terminated by adding 6 mL of ethanol. Finally, the suspension was centrifuged at 10,000 rpm for 15 min, and the precipitate was washed with deionized water and dialyzed against deionized water. Finally, 0.68 g of bright white TOCNFs were obtained after being freeze-dried.

### 2.3. Morphology Analysis

The morphology of U-cellulose, CNFs, and TOCNFs was checked by scanning electron microscopy (SEM, SU8010/PP3010T, Hitachi, Tokyo, Japan), and all the samples were sprayed with gold to improve the image resolution. Then, the CNFs suspension (0.001 wt%) was dropped onto mica substrate and imaged by atomic force microscope (AFM, AFM5500, Hitachi, Japan) after natural air drying. TOCNFs suspension (1 mg/mL) was dropped onto the carbon film and checked by transmission electron microscope (TEM, JEM-2100UHR, JEOL, Tokyo, Japan) at the acceleration voltage of 200 kV. Image analysis software “Image J” (Image J, 1.50i, 64-bit) was used to measure the length and diameter of 50 randomly selected valid samples on the images.

### 2.4. Characterization of U-Cellulose, CNFs and TOCNFs

The functional group of all the samples were determined by Fourier transform infrared (FT−IR) spectrometer (Spectrum Two, PerkinElmer, Waltham, MA, USA). Dried potassium bromide was used as a matrix by mixing with freeze-dried sample powder in a weight ratio of 1:40, which was fully ground in a mortar and pressed into flakes. Data were collected in the spectral range of 4000–400 cm^−1^. The crystallinities of CNFs and TOCNFs were analyzed by X-ray diffraction (XRD-7000s, Shimadzu, Kyoto, Japan). The equipment was operated at 40 mA current, 40 kV voltage using Cu Kα as a radiation source with diffraction angle from 10–60° and scanning rate of 4°/min. The crystallinity index (CrI %) was calculated using Segal equation:

CrI (%) = [(I_002_ − I_Am_)/I_002_] × 100% (1)

where I_002_ was the peak intensity at plane (002) (2θ = 22.6°), and I_Am_ was the minimum peak intensity between plane (002) and (011) (2θ = 18°) [23].

A thermal analyzer (Thermal Analyzer Q500, TA Instruments, New Castle, DE, USA) was used for thermogravimetric analysis (TGA) of the samples. Before the experimental analysis, CNFs and TOCNFs samples were placed in a 60 °C oven and dried for 24 h in advance. About 5 mg of the samples were placed in an alumina crucible and heated from room temperature to 600 °C at a heating rate of 10 °C/min under a nitrogen atmosphere. The flow rate of nitrogen was 60 mL/min.

The particle size of the TOCNFs samples in deionized water (10 mg/mL) was measured after ultrasonic treatment at 0, 150, 250, 350, 450, and 550 W using an ultrasonic cell disrupter (SCIENTZ-950E, Scientz, Ningbo, China). The length of the TOCNFs treated with ultrasonic cell disrupter was measured by a 3D light scatterer (3D LS, LS Instruments AG, Fribourg, Switzerland). A total of 1 mL of suspension was added to the sample bottle and placed in the adapter. The dynamic light laser wavelength was 633 nm. The ζ−potential of the suspensions was measured by Malvern potentiometer (ZETASIZER 3000HSA, Malvern Instruments Ltd., Malvern, UK). All experimental measurements were repeated three times.

### 2.5. Preparation of Pickering Emulsions Stabilized with TOCNFs

The freeze-dried TOCNFs samples (0.9 g) treated with 450 W ultrasound were dispersed into 10 mL deionized water at a concentration of 0.9 wt%. Subsequently, 2.5 mL sunflower oil was added to 10 mL of the suspensions to maintain an oil/water ratio of 2:8. Pickering emulsion was prepared at different ultrasonic power (0, 150, 250, 350, 450, and 550 W).

### 2.6. Influencing Factors of Pickering Emulsion

#### 2.6.1. Effect of TOCNFs Concentrations on Pickering Emulsion

Different masses of freeze-dried TOCNFs were added to 10 mL deionized water to form suspensions with different mass fractions (0.15 wt%, 0.30 wt%, 0.60 wt%, 0.90 wt%, 1.20 wt%, and 1.50 wt%), respectively. Then, sunflower oil was added as oil phase, accounting for 20% (*v*/*v*) of the overall volume. Pickering emulsion was prepared by ultrasonic treatment for 10 min at 450 W.

#### 2.6.2. Effect of pH and Ionic Strength on Pickering Emulsion

A volume of 10 mL suspensions containing 0.9 wt% of TOCNFs was adjusted to different pH values (2, 4, 6, 8, 10, and 12), respectively, before mixing with sunflower oil. The mixed suspension was treated with ultrasound at 450 W for 10 min. The influence of ionic strength on Pickering emulsion was investigated by adding NaCl powder to each sample until the mass fractions of NaCl were 0.2 wt%, 0.4 wt%, 0.6 wt%, 0.8 wt%, 1.0 wt%, and 2.0 wt% under the optimum pH condition.

### 2.7. Characterization of Pickering Emulsions

#### 2.7.1. Morphology Analysis of Pickering Emulsions

The microstructure of emulsified layer was checked by SEM, while the droplet size and distribution of Pickering emulsions stabilized with TOCNFs were examined by inverted fluorescence microscope (Eclipse TI-U, Nikon, Tokyo, Japan). In order to obtain better imaging, 200 μL of emulsion was mixed with 5 μL of sodium fluorescein staining solution (5 mg/mL) for 15 min. The 10 μL stained emulsion was observed under a 20-fold microscope at collecting emission wavelength of 520–530 nm.

#### 2.7.2. Droplets Size Determination and Emulsion Index (EI)

The average diameter of 100 random droplets was analyzed by “Image J” as well as the emulsion index (EI) according to the following equations:

EI (%) = H_E_ ⁄ H_T_ × 100% (2)

where H_E_ (cm) was the emulsion layer height, and H_T_ (cm) was the total emulsion height in the tube.

### 2.8. Preparation of AXT-Loaded Pickering Emulsion

The powder of AXT crude (5 g) was dissolved in 100 mL sunflower oil and stirred in dark for 24 h, followed by centrifugation at 3000 rpm for 15 min to remove the insoluble impurities [24]. TOCNFs (0.9 wt%) was dispersed in deionized water, and the pH of the aqueous phase was adjusted to 4.0. After adding the AXT-loaded sunflower oil to the TOCNFs suspensions at a ratio of 2:8, ultrasonic treatment was conducted for 10 min at 450 W power. The prepared AXT-loaded Pickering emulsion was stored at 4 °C from light for further use.

### 2.9. Stability Analysis of AXT-Loaded Pickering Emulsion

The sample bottle contained of AXT-loaded Pickering emulsion was wrapped in tin foil and placed in an oven at different temperature (25, 50, 75, 100, and 125 °C) with hot air circulation for 1.5 h. The samples were then cooled for 30 min at room temperature to investigate the thermal stability of the emulsion. The storage stability of emulsion was tested by placing the samples at room temperature in the dark for 14 days.

### 2.10. Encapsulation Efficiency (EE)

UV-Vis spectrophotometer (Lambda 35, PerkinElmer, Waltham, MA, USA) was used to measure the encapsulation efficiency of the emulsion after thermal stability and storage stability according to the previous method with slight modification [25,26]. In brief, the above AXT-loaded Pickering emulsions were mixed with hybrid organic solvent (dichloromethane: methanol = 2:1 (*v*/*v*) in a ratio of 1:9. After mixing, the samples were centrifuged at 10,000 rpm for 20 min. The optical density (OD) value of the organic phase layer was measured at 480 nm by UV-Vis spectrophotometer. Similarly, the OD value of the oil phase was measured in the same way. AXT concentration (μg/mL) was calculated by the AXT standard curve (Figure S1, y = 0.3189x + 0.0153, R^2^ = 0.9992, *n* = 7, 0.5–5.0 μg/mL, limit of detection (LOD) = 0.377 μg/mL, limit of quantification (LOQ) = 1.141 μg/mL) obtained by dissolving standard AXT in organic solvents using pure organic solvent as a blank control. Finally, the EE (%) of AXT was calculated as follows [27]:

EE (%) = C_1_ ⁄ C_0_ × 100% (3)

where C_1_ (μg/mL) was the AXT concentration in Pickering emulsion, and C_0_ (μg/mL) was the initial concentration of AXT in the oil phase.

### 2.11. In Vitro Simulated Digestion

The release of FFAs and bioaccessibility of AXT were measured using *in vitro* simulated digestion model according to the previous method by Zou et al. [28] with slight modification. The AXT loaded in both sunflower oil and Pickering emulsion were diluted with 5 mM PBS buffer solution to ensure the oil occupied the same mass fraction in the buffer (2 wt%). The samples were placed in an incubator shaker (ZWY-211B, Zhicheng, China) at 37 °C for further use.

Simulated gastric fluid (SGF) was prepared by adding 0.2 wt% NaCl, and 3 mg/mL pepsin into deionized water, and pH was adjusted to 2.5 by adding 1 mol/L of HCl. The prepared SGF (7.5 mL) was placed in the incubator shaker at 37 °C for 15 min, then mixed with the initial diluted sunflower oil or Pickering emulsion in equal volume and stirred at 100 rpm for 2 h [29].

Simulated intestinal fluid (SIF) was composed of 2.25 mL salt solution (218.7 mg/mL NaCl, 27.66 mg/mL CaCl_2_), 3.75 mL lipase suspension (24 mg/mL), 3.75 mL trypsin suspension (24 mg/mL), and 5.25 mL bile salt extract (Bovine) (54 mg/mL). The pH of the SGF digested mixture was adjusted to 7.0 and mixed with SIF in a ratio of 1:2. At a suitable time interval (5 min), the pH of the reaction system was maintained at 7.0 by adding 0.1 M NaOH. Then the FFAs (%) was calculated as follows:

FFAs (%) = (V_NaOH_ × C_NaOH_ × M_oil_)/(2 m_oil_) × 100% (4)

where V_NaOH_ (L) was the volume of NaOH added during the titration, C_NaOH_ (mol/L) was the molar concentration of the titrated sodium hydroxide solution, M_oil_ (g/mol) was the molecular weight of the oil used, and m_Oil_ (g) was the total mass of the oil added before digestion.

A certain volume of digesta was taken and centrifuged at 10,000 rpm for 40 min. After centrifugation, the intermediate micellar layer was taken, and the concentration of AXT was measured according to the same extraction method as Section 2.10. Finally, the bioaccessibility (%) of AXT was calculated as follows:

Bioaccessibility (%) = C_micelle_/C_initial_ × 100% (5)

where C_micelle_ (μg/mL) was AXT concentration in micellar layer, and C_initial_ (μg/mL) was the concentration in initial emulsion or oil phase [24].

### 2.12. Statistical Analysis

Data were analyzed by SPSS software (version 18.0), and one-way analysis of variance (ANOVA) was performed for statistical analysis. When *p* < 0.05, data were considered statistically significant. All data are presented as mean ± standard deviation (*n* = 3).

## 3. Results and Discussion

### 3.1. Preparation of U-Cellulose, CNFs, and TOCNFs

**Figure 1:**
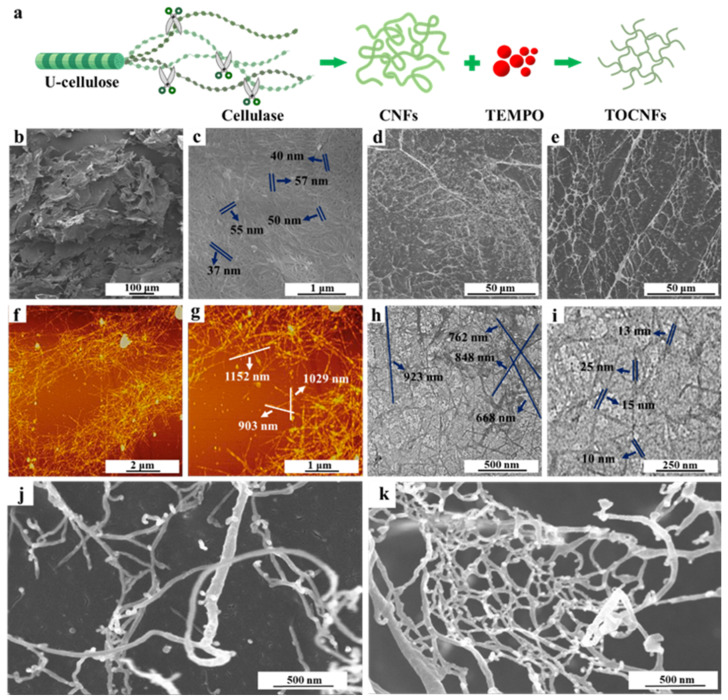
Schematic illustration for the preparation of nanocellulose (**a**). SEM images of U-cellulose (**b**,**c**), CNFs (**d**), and TOCNFs (**e**); AFM images of CNFs (**f**,**g**); TEM images of TOCNFs (**h**,**i**); amplified SEM images of CNFs (**j**) and TOCNFs (**k**).

The schematic illustration for the preparation of nanocellulose is shown in Figure 1a. The U-cellulose was prepared via alkali and bleaching treatments of *Undaria pinnatifida* powder to remove hemicellulose and lignin with a final yield of 56.80%. Then, the CNFs were prepared by enzymatic hydrolysis method to give a production yield of 54.33%, and TOCNFs were obtained through oxidization by TEMPO with a yield of 68.16%. The crystallinity and dimensions of different samples were summarized in Table S1. As shown in Figure 1b, the U-cellulose exhibits a dense flaky structure covered by a large number of winding micron filament (Figure 1c). The diameter of U-cellulose was 47.01 ± 11.85 nm, and the length as well as aspect ratio could not be calculated by software due to the tight filament winding. Figure 1d,e shows that the filamentous network of CNFs and TOCNFs become loose. The normal and enlarged AFM images indicate that the average diameter of CNFs is 1036.95 ± 111.89 nm, while the average diameter is 26.22 ± 5.02 nm (Figure 1f,g). The TOCNFs exhibit a needle-like shape, with the average length and diameter reduced to 773.52 ± 91.81 and 13.52 ± 3.3 nm, respectively (Figure 1h,i). Although both the length and diameter of TOCNFs was reduced, it was noteworthy that the TOCNFs had a higher aspect ratio of 56.79 (Table S1). The enlarged SEM images of CNFs and TOCNFs in Figure 1j,k displayed that the spatial structure of CNFs was disorderly arranged, and the filamentous CNFs were intertwined. The TOCNFs formed a stable spatial structure, and nanofibers were tightly packed, yielding a network structure. This was because the carboxylic acid group introduced after oxidation replaced the hydroxyl group, which weakened the hydrogen bond force of cellulose and hindered the adhesion between cellulose [30]. The resulting network structure might stabilize the oil droplets in the void and prevent aggregation of oil droplets, thus forming a stable Pickering emulsion in the subsequent applications.

### 3.2. FT−IR, XRD and TGA Analysis of U-Cellulose, CNFs, and TOCNFs

**Figure 2:**
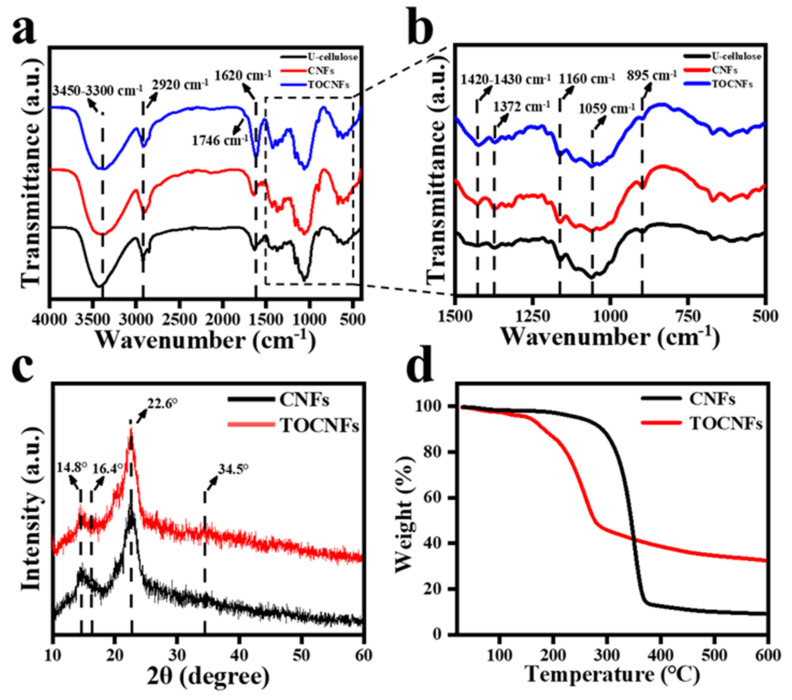
FTIR spectra of U-cellulose, CNFs, and TOCNFs (**a**); amplification of FTIR spectra in the 1500–500 cm^−1^ region (**b**). XRD spectra of CNFs and TOCNFs (**c**). TGA curves of CNFs and TOCNFs (**d**).

The FT−IR spectra of U-cellulose, CNFs, and TOCNFs in Figure 2a,b show the presence of peak at 3450–3300 cm^−1^ due to the stretching vibration of O-H, and the intensity of the peak of TOCNFs decreased due to the reduction of hydroxyl group in the oxidation process compared with U-cellulose and CNFs. The peak at 2900–2800 cm^−1^ was assigned to the stretching vibration of C-H [31]. It was observed from the FT−IR spectra of TOCNFs that a small absorption peak appeared at 1746 cm^−1^, indicating that the hydroxyl group was successfully oxidized to carboxyl group. Compared with U-cellulose and CNFs, the intensity of the peak at 1620 cm^−1^ assigned to the O-H of water in TOCNFs increased, which proved its ability binding to water was stronger [32]. The peak at 1735 cm^−1^ corresponded to the stretching of C=O bonds in the carbonyl, ester, and acetyl groups of hemicellulose and lignin [33], and the band of 1530 cm^−1^ corresponded to aromatic skeleton (C=C) vibration of lignin [34]. Moreover, the peak at 1270 cm^−1^ was related to the C-O stretching of aryl groups in lignin [35]. Notably, these peaks were not observed in the FT−IR spectra, indicating that hemicellulose and lignin had been successfully removed during the extraction process. The peaks in the range of 1420–1430 cm^−1^ and 1372 cm^−1^ in all samples were related to the symmetric bending of CH_2_ and the bending vibrations of C-H and C-O groups, respectively [36,37], which were the typical structure of cellulose. The C-O-C asymmetric stretching of cellulose was indicated by the absorption peak near 1160 cm^−1^ [38]. Compared with the U-cellulose, the peak intensity of CNFs and TOCNFs significantly increased, indicating an increase in nanocellulose content. The absorption peak near 1059 cm^−1^ could be ascribed to the vibration of C-O-C pyran ring skeleton in cellulose [39], and the absorption peak near 895 cm^−1^ was related to the β-glucoside bond of glucose in cellulose. It can be seen from the FT−IR spectra that impurities had been effectively removed during the preparation of cellulose. The cellulase hydrolysis and TEMPO oxidation were both somewhat mild chemical treatments, which did not destroy the original structure of cellulose.

XRD was further used to observe the difference in crystallinity between CNFs and TOCNFs (Figure 2c) In the XRD patterns of CNFs and TOCNFs, there were main intensity peaks at 2θ = 14.8°, 16.4°, 22.6°, and 34.5°, indicating that both CNFs and TOCNFs had typical cellulose I crystal structure [33]. According to the Segal equation, the CNFs had a high crystallinity of 73.1%, which was contributed to the removal of amorphous regions such as hemicelluloses by alkali, bleaching, and cellulase treatment while retaining the crystalline regions of cellulose [37]. After TEMPO oxidation, the crystallinity of TOCNFs was 69.6%, which was due to the selectivity of the oxidation process. The formation of amorphous glucuronic acid unit mainly occurred on the crystalline surface of CNFs [40], so the overall structure of the crystalline area was not damaged, and TOCNFs still maintained a relatively high degree of crystallinity.

TGA curves of CNFs and TOCNFs in Figure 2d revealed that the degradation process of CNFs began at about 315.9 °C. The maximum thermal degradation temperature was 346.8 °C, and the carbon residue rate was 9.08%. Compared to the CNFs, the TOCNFs degraded at about 100 °C due to the degradation of bound water and had an obvious thermal degradation at 209.5 °C. The final carbon residual rate of TOCNFs was 32.83%, and the maximum thermal degradation temperature was 263.6 °C. According to the previous report, the change in thermal degradation temperature might also be related to the change in the crystallinity of TOCNFs [30]. The TOCNFs became more sensitive to the temperature change after the treatment of TEMPO.

### 3.3. Particle Size and ζ−Potential of U-Cellulose, CNFs, and TOCNFs

**Figure 3:**
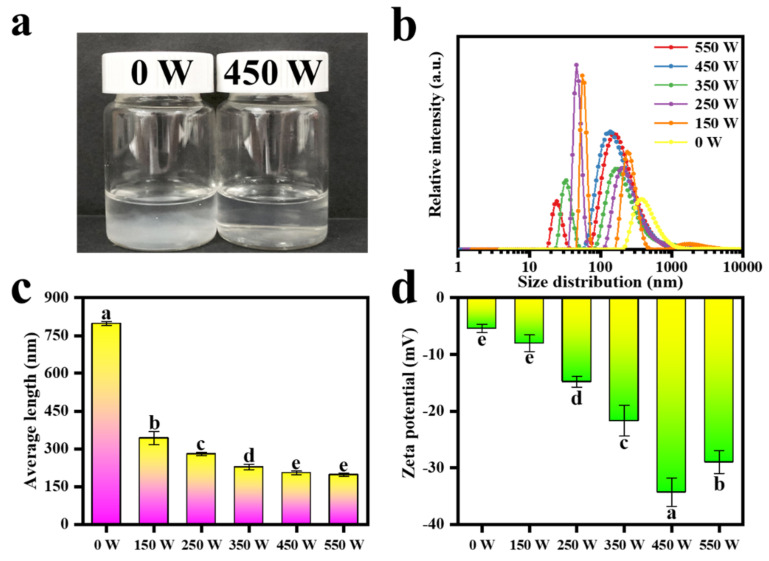
Photograph of TOCNFs suspension treated at 0 W and 450 W ultrasonic power (**a**). The size distribution diagram of TOCNFs at different ultrasonic power (0, 150, 250, 350, 450, and 550 W) (**b**). Average length (**c**) and ζ−potential (**d**) diagram of TOCNFs at different ultrasonic power (0, 150, 450, 250, 350, 450, and 550 W). Different letters of a, b, c, d and e show significant differences.

The size distribution and average length of TOCNFs were obtained under the same ultrasonic time and different power treatments (Figure 3). Compared with the untreated sample, the suspension became almost transparent after 450 W ultrasonic treatment (Figure 3a). This meant that the long TOCNFs were cut into short fibers, which showed a better dispersion in the aqueous phase. Figure 3b, c exhibits the DLS results of the TOCNFs under different powers of ultrasonic treatment. There is a decrease trend for the average length for the TOCNFs along with the increase of the ultrasonic power. It is noteworthy that the length of untreated TOCNFs was 798.22 ± 8.28 nm. After 150 W ultrasonic treatment, the size of TONCFs significantly reduced to 342.79 ± 25.44 nm due to the large amount of energy generated by acoustic cavitation. With the increasing of the ultrasonic power from 150 W to 350 W, the size of the TOCNFs displayed bimodal distribution continuously. While at 450 W, the size distribution presented a good single-peak distribution with an average size of 204.19 ± 3.05 nm. However, when the TONCFs was treated at 550 W, the average size did not change significantly (*p* > 0.05). Besides, higher ultrasonic power would consume excessive energy and produced two populations of size distribution again. Moreover, ζ−potential was a good indicator to evaluate the physical stability of the suspension system. Figure 3d shows the potential diagram of TOCNFs suspension under different treatment conditions. As expected, the suspension treated with 450 W exhibited a minimum potential of −34.3 ± 2.51 mV. This could be attributed to the increased contact area between TOCNFs and oxygen by ultrasonic treatment, resulting in more negative charge on the surface of the nanoparticles [41]. Therefore, 450 W was considered as the optimal ultrasonic treatment condition to obtain the nanocellulose suspension with the best stability, and the TOCNFs produced by 450 W ultrasonic power were further used as the aqueous phase of Pickering emulsions.

### 3.4. Effect of Ultrasonic Power on Pickering Emulsions

**Figure 4:**
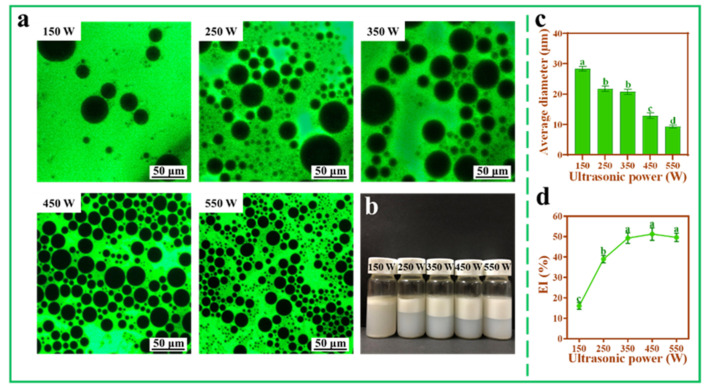
Inverted fluorescence microscopy images (**a**), photograph (**b**), average diameter (**c**), and EI% (**d**) of Pickering emulsions at different ultrasonic power (150, 250, 350, 450, and 550 W, pH = 7, ω(NaCl) = 0, ω(TOCNFs) = 0.9 wt%). Different letters of a, b, c and d show significant differences.

The influence of ultrasonic power on the Pickering emulsions was investigated under the condition of fixed mass fraction of TOCNFs (0.9 wt%). It could be seen from the distribution of dyeing area that the Pickering emulsion formed was oil-in-water type. At 150 W ultrasonic power, the EI% was very low with a little amount of oil precipitation and thus could not form stable Pickering emulsions (Figure 4b,d). When the power increased from 250 W to 550 W, the average diameter of the droplets decreased from 21.71 ± 0.89 to 9.29 ± 0.43 μm (Figure 4c). Similarly, with the increase of ultrasonic power, the emulsification layer gradually became higher, and the droplet size tended to be relatively uniform (Figure 4a,b). This was because TOCNFs could be easily adsorbed at the oil–water interface under high-power conditions. However, compared with the samples under 450 W ultrasonic treatment, there was no significant difference (*p* > 0.05) in EI% at 550 W even though the diameters of the droplets were still decreasing (Figure 4c,d). Based on the results in Figure 3b,c, it could be easily found that under 550 W ultrasonic intensity, smaller TOCNFs appeared, which were adsorbed to the oil/water interface and encapsulated smaller oil droplets as reported in previous work by Ni et al. [35].

### 3.5. Effect of TOCNFs Concentrations on Pickering Emulsions

**Figure 5:**
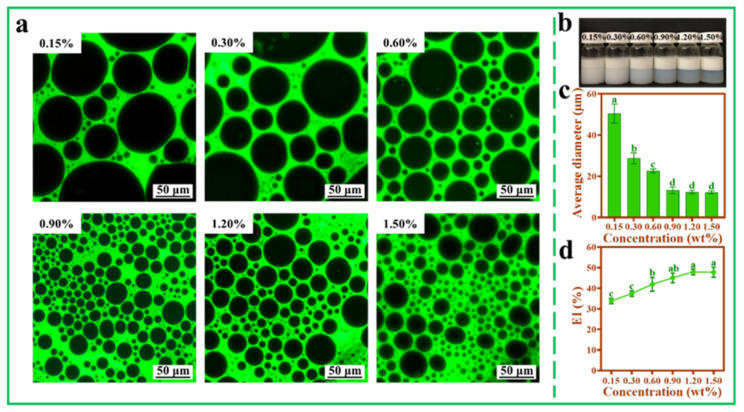
Inverted fluorescence microscopy images (**a**), photograph (**b**), average diameter (**c**), and EI% (**d**) of Pickering emulsions at different TOCNFs concentrations (0.15 wt%, 0.30 wt%, 0.60 wt%, 0.90 wt%, 1.20 wt%, and 1.50 wt%, pH = 7, ω(NaCl) = 0, 450 W). Different letters of a, b, c and d show significant differences.

The effects of different concentrations of TONCFs on Pickering emulsions were investigated at a fixed ultrasonic power of 450 W (Figure 5a). Overall, even at lower TOCNFs concentrations, an emulsion layer higher than the oil phase could be obtained (Figure 5b). When the concentration was lower (0.15%), the emulsion droplet size was larger, and the size distribution was not uniform (Figure 5a). As the concentrations increased, the TOCNFs could be better dispersed at the oil–water interface, thus effectively covering the surface of oil droplets and enhancing the anti-aggregation ability between droplets [42,43]. With the concentration of TOCNFs increased to 0.9 wt%, the thickness of the emulsion layer increased (Figure 5b), the droplet size distribution became uniform, and the size of large droplets was reduced (Figure 5a). When the concentration of TOCNF increased from 0.9 wt% to 1.50 wt%, the average diameter of droplets and EI% did not change significantly (*p* > 0.05) (Figure 5c,d). Therefore, excessive TOCNFs did not contribute to the encapsulation of oil droplets. In this work, droplet size distribution was considered as an important criterion for emulsions evaluation. Therefore, 0.9 wt% TOCNFs was further used to investigate the effect of pH and ion strength on Pickering emulsions in subsequent experiments.

### 3.6. Effect of pH and Ionic Strength on Pickering Emulsions

**Figure 6:**
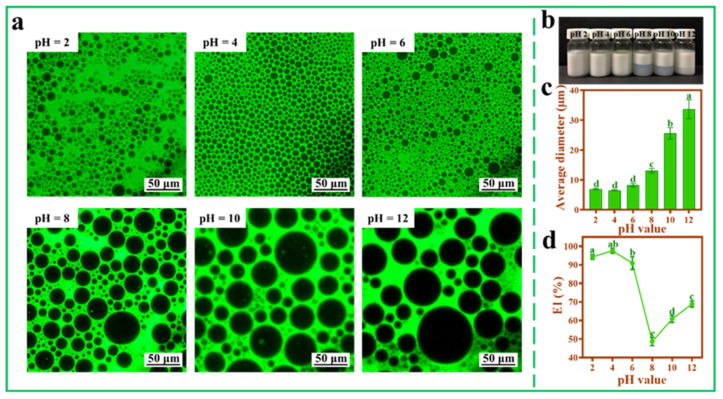
Inverted fluorescence microscopy images (**a**), photograph (**b**), average diameter (**c**), and EI% (**d**) of Pickering emulsions at different pH (2, 4, 6, 8, 10, and 12; ω(NaCl) = 0, ω(TOCNFs) = 0.9 wt%, 450 W). Different letters of a, b, c and d show significant differences.

Figure 6 shows that the pH value of TOCNFs imposed a great influence on Pickering emulsions. The smallest droplets (6.52 ± 0.20 μm) could be obtained without aqueous phase separation when pH was 4.0 (Figure 6b,c). In the acidic environment, the size of emulsion droplets was small, and the size distribution was relatively uniform (Figure 6a). Therefore, a lower pH could be beneficial to increase the negative surface charge of TOCNFs, which was coincided with Li’s report, who prepared Pickering emulsions using *Miscanthus floridulus* straw nanofibers and found that a lower pH value led to a smaller droplet [44]. It was worth noting that when pH rose from 8 to 12, the EI% increased (Figure 6d), the reason of which might be due to the fact that aggregation of emulsion droplets increased the average diameter and made the emulsion layer higher. A similar result was reported when kelp nanocellulose was applied in preparing Pickering emulsions [21].

The photographs and microscope images at different concentrations of NaCl are displayed in Figure S2. The average diameter of droplets increased, and the Pickering emulsions gradually showed hydrophilic and hydrophobic layers with the increase of NaCl content (Figure S2a,b). When the concentration of NaCl increased to 0.4 wt%, the EI% decreased significantly (Figure S2d). Moreover, as the concentration of NaCl reached 2 wt%, the average diameter of droplets reached 36.21 ± 3.10 μm (Figure S2c). Therefore, the increase of ionic strength was not conducive to the stable formation of the emulsions. This was because sodium, with a positive charged in the electrolyte, would gather around TONCFs and neutralize the negative charge on the surface of TOCNFs, leading to the reduction of charge repulsion between the droplets, thus making the droplets’ aggregation and size increase [45]. The stable Pickering emulsion turned to two layers eventually in the presence of strong ionic intensity of NaCl.

### 3.7. Morphology of Emulsion Droplets

**Figure 7:**
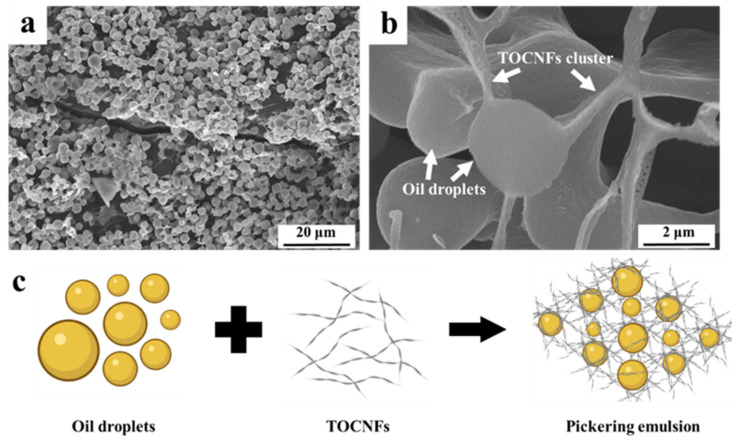
SEM of Pickering emulsions stabilized with TOCNFs (**a**,**b**); schematic diagram of Pickering emulsions stabilized with TOCNFs (**c**).

The morphology of the oil droplets and distribution of TOCNFs in Pickering emulsions prepared under the condition of pH = 4 and TOCNFs concentration of 0.9 wt% was characterized with SEM technology (Figure 7). Spherical particles with a size around 2 μm were observed with a threadlike structure connected each other (Figure 7a). The TOCNFs probably formed this filamentous structure adsorbed onto the oil–water interface to prevent droplets from aggregation. The amphiphilic TOCNFs mainly existed in the water phase, connecting the droplets, and eventually formed a stable network within Pickering emulsions (Figure 7b). This process was further explicated by the schematic illustration in Figure 7c. The TOCNF-supported network structure was formed under ultrasonic treatment in the oil–water interface, thus supporting the emulsions system. The Pickering emulsions prepared by nanocellulose from lemon seeds had the same morphological characteristics [46]. Therefore, the SEM result further confirmed that the TOCNFs were applicable in fabricating a stable Pickering emulsion with regular network connected with the nanofiber.

### 3.8. Thermal Stability of AXT-Loaded Pickering Emulsions

**Figure 8:**
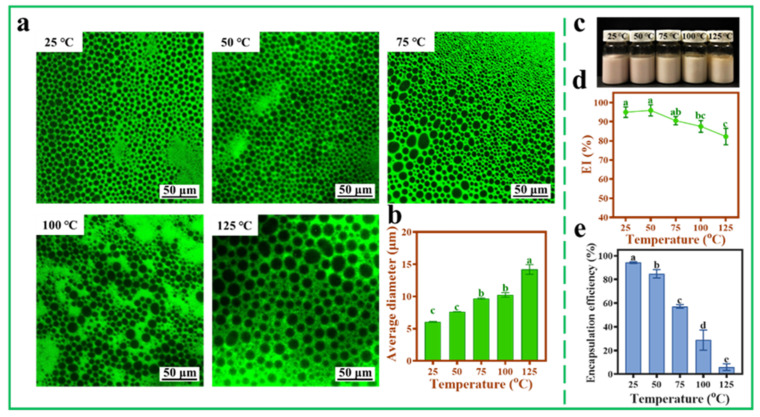
Inverted fluorescence microscopy images (**a**), average diameter (**b**), photograph (**c**), EI% (**d**), and EE% (**e**) of AXT-loaded Pickering emulsions at different temperatures (25, 50, 75, 100, and 125 °C, pH = 4, ω(NaCl) = 0, ω(TOCNFs) = 0.9 wt%, 450 W). Different letters of a, b, c, d and e show significant differences.

In order to further explore the practical application potential of Pickering emulsions stabilized with TOCNFs, the hydrophobic AXT was loaded into the Pickering emulsions, and the thermal stability and storage stability of the AXT-loaded Pickering emulsions were tested at different temperatures. Compared with pH and ionic strength, temperature also had influence on droplet size, which increased with the increase of the temperature. The average diameter of the droplets increased from 6.07 ± 0.05 μm to 14.19 ± 0.76 μm as the temperature increased from 25 °C to 125 °C (Figure 8a,b). The droplets also maintained a spherical shape even at 125 °C, suggesting a good thermal stability of nanocellulose. From the photograph of the emulsions, it could be seen that the color changed from pink to yellow with the increase of heat treatment temperature from 25 to 125 °C (Figure 8c). Under 125 °C treatment, a small amount of oil was precipitated from the upper layer of the emulsions, and the EI% was reduced to 82.21 ± 4.29% (Figure 8c,d). This was because the high temperature would weaken the forces of the nanocellulose networks, leading to the collapse of droplets and reforming of larger-sized particles [35]. Moreover, when the temperature increased from 25 °C to 75 °C, the EE% of AXT decreased from 94.24 ± 0.73% to 57.23 ± 1.42% (Figure 8e). The retention rate of AXT was better than the previous study by Boonlao et al., in which the emulsions prepared by whey protein isolate and xanthan gum were about 30% at 70 °C [29]. Generally, the storage temperature for the AXT encapsulation is within room temperature, and the EE% of AXT can be well maintained more than 80% even at a relative higher temperature in summer below 50 °C.

### 3.9. Storage Stability of AXT-Loaded Pickering Emulsions

Storage stability was also an important property for AXT-loaded Pickering emulsions. The emulsions were stored at 25 °C, away from light for 14 days. As shown in Figure S3, with the extension of storage time, the color of the emulsions was faded to a certain extent. Moreover, as can be seen from the results in the Table 1, after 14 days of storage, the EI% of the emulsions decreased from 94.93 ± 2.76% to 86.39 ± 5.16%. The EE% of AXT decreased to 80.64 ± 7.44%, which was not significantly different from the initial EE% (*p* > 0.05), indicating that astaxanthin has a high retention rate. Shen et al. prepared emulsions by whey protein isolates that showed comparable AXT retention after 14 days of storage, which showed better stability than the other emulsion systems tested [47]. The storage results proved that the small size of the emulsion droplets could resist gravity separation and aggregation, thus effectively preventing the decomposition of AXT [24].

### 3.10. Release of FFAs and Bioaccessibility Analysis

**Figure 9:**
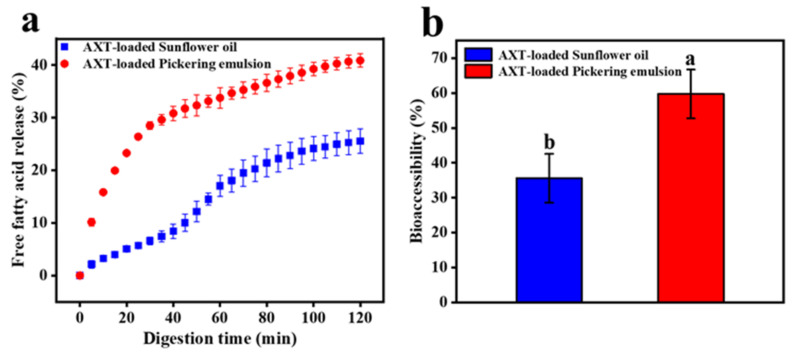
The release of FFAs (**a**) and bioaccessibility of AXT (**b**) of AXT-loaded Pickering emulsions and sunflower oil in simulated intestinal fluid. Different letters of a and b show significant differences.

The release amount of FFAs could reflect the digestion status of the emulsions. As shown in Figure 9a, after *in vitro* digestion, the release rate of FFAs in AXT-loaded Pickering emulsions increased sharply in the first 40 min, which was significantly higher than that of sunflower oil. The total release of FFAs from AXT-loaded Pickering emulsions and sunflower oil was 40.88 ± 1.88% and 20.56 ± 2.28%, respectively, after 120 min *in vitro* digestion. The digestion of FFAs was thought to be a long process; due to the small droplet size of Pickering emulsions stabilized with TOCNFs, oil digestion was accelerated by increasing the surface area for contact with lipase and trypsin [48]. On the contrary, the thick, interfacial layer of sunflower oil inhibited trypsin digestion and made it difficult to remove the long FFAs chains from the oil surface [24,49]. After digestion, AXT content of digesta in the two samples was compared. AXT was a lipophilic molecule with poor water solubility, so it had to be dissolved in the mixed micellar layer before it could be digested and absorbed [28]. Therefore, the bioaccessibility of AXT was determined by measuring AXT content before and after digestion. The bioaccessibility of AXT in Pickering emulsions and sunflower oil was 59.75 ± 6.99% and 35.62 ± 6.98%, respectively (Figure 9b). It could be seen that the release amount of AXT in the emulsions was much higher than that in sunflower oil. Due to the faster and higher release rate of FFAs in the emulsions, a higher proportion of digested lipids resulted in the formation of more mixed micelles with AXT molecules. Therefore, Pickering emulsions stabilized with TOCNFs could improve the bioaccessibility of lipophilic active molecules, such as AXT.

## 4. Conclusions

In this study, AXT-loaded Pickering emulsions were successfully prepared with TEMPO-modified CNFs extracted from *Undaria pinnatifida* via ultrasonic method. The Pickering emulsions with relatively uniform droplet size were obtained under the ultrasonic power of 450 W. Emulsion stability tests showed that when the concentration of TOCNFs was 0.9 wt%, pH was 4.0, and no NaCl was added, the emulsions were the most stable without any oil-phase precipitation. SEM images demonstrated that TOCNFs were adsorbed on the oil–water interface so that the oil droplets were coated in the gaps in the cellulose network, which realized the stability of the oil droplets. Notably, the Pickering emulsions stabilized with TOCNFs had a good protective effect on AXT at high temperature and exhibited excellent storage stability. *In vitro* digestion showed that AXT-loaded Pickering emulsions displayed higher bioaccessibility for AXT. These results indicated that Pickering emulsions stabilized with nanocellulose have good application potential as stable carriers for AXT and could offer valuable insights for developing advanced, functional foods.

## Figures and Tables

**Table 1:** EE%, EI%, and the average diameter of AXT-loaded Pickering emulsions stored for 1 day, 7 days, and 14 days.

AXT-Loaded Pickering Emulsions	EE (%)	EI (%)	Average Diameter (μm)
1stday	94.24 ± 0.73 ^a^	94.93 ± 2.76 ^a^	6.07 ± 0.32 ^a^
7th day	86.65 ± 8.71^a^	87.92 ± 3.90 ^ab^	6.31 ± 0.39 ^b^
14th day	80.64 ± 7.44 ^a^	86.39 ± 5.16 ^b^	7.09 ± 0.04 ^c^

Different superscript letters represent significant difference (*p* ≤ 0.05).

## Data Availability

The datasets generated for this study are available on request to the corresponding author.

## Supplementary Materials

The following supporting information can be downloaded at: https://www.mdpi.com/article/10.3390/foods11060876/s1, Table S1: Yield, length, diameter, and aspect ratio of U-cellulose, CNFs, and TOCNFs; Figure S1: The AXT standard curve; Figure S2: Inverted fluorescence microscopy images (a), photograph (b), average diameter (c), and EI% (d) of Pickering emulsions at different NaCl concentrations (0.20 wt%, 0.40 wt%, 0.60 wt%, 0.80 wt%, 1.0 wt%, and 2.0 wt%; ω(TOCNFs) = 0.9 wt%, pH = 4, 450 W); Figure S3: Photographs of AXT-loaded Pickering emulsions stored for 1 day, 7 days, and 14 days.
