# Supplementary material for: Preparation and Evaluation of Undaria pinnatifida Nanocellulose in Fabricating Pickering Emulsions for Protection of Astaxanthin
Source: Foods. 2022 Mar 18;11(6):876. doi: 10.3390/foods11060876 (PMC8956011; doi:10.3390/foods11060876)
Supplement: Supplementary file 1 [file foods-11-00876-s001.zip › foods-1611405-supplementary.pdf]

foods

Supplementary data

Preparation and Evaluation of *Undaria pinnatifida* Nanocellulose in Fabricating Pickering Emulsions for Protection of Astaxanthin

Yu Li [1,2,3], Siyuan Fei [1,2,3], Deyang Yu [1,2,3], Lijuan Zhang [1,2,3], Jiaxuan Li [1,2,3], Ronggang Liu [1,2,3] and Mingqian Tan [1,2,3,*]

[1] Academy of Food Interdisciplinary Science, School of Food Science and Technology, Dalian Polytechnic University, Qinggongyuan 1, Ganjingzi District, Dalian 116034, China; liyu06190908@163.com (Y.L.); 13470319363@163.com (S.F.); yudy0411@163.com (D.Y.); zhanglijuan202202@163.com (L.Z.); nancylee98@126.com (J.L.); lrg19950427@163.com (R.L.)
[2] National Engineering Research Center of Seafood, Dalian Polytechnic University, Dalian 116034, China
[3] Collaborative Innovation Center of Seafood Deep Processing, Dalian Polytechnic University, Dalian 116034, China
* Correspondence: mqtan@dlpu.edu.cn; Tel.: +86-411-8631-8657

Table S1. Yield, length, diameter and aspect ratio of U-cellulose, CNFs and TOCNFs.

Sample	U-cellulose	CNFs	TOCNFs
Yield (%)	56.80 ± 7.42[ab]	54.33 ± 6.03[b]	68.16 ± 4.43[a]
Average length (nm)	-	1036.95 ± 111.89[a]	773.52 ± 91.81[b]
Average diameter (nm)	47.01 ± 11.85[a]	26.22 ± 5.02[b]	13.52 ± 3.30[b]
Aspect ratio	-	39.98 ± 3.45[b]	56.79 ± 8.59[a]

Different superscript letters represent significant difference ($p < 0.05$).

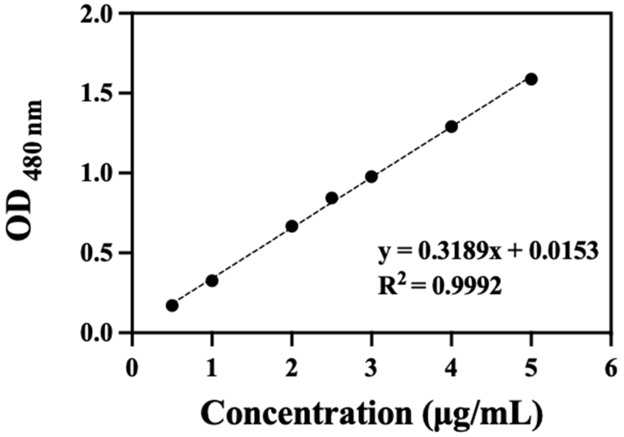

Figure S1. The standard curve of AXT.

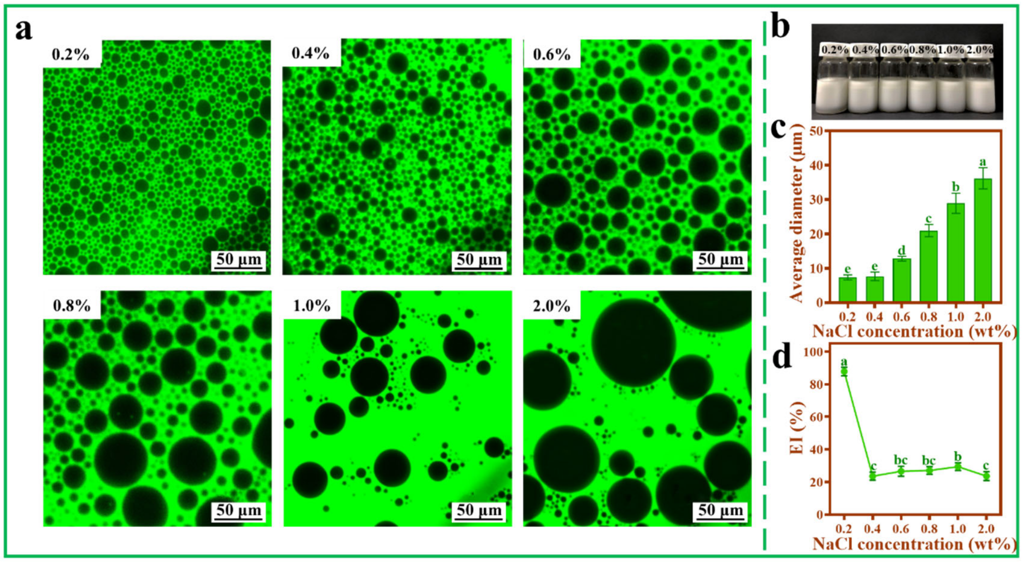

Figure S2. Inverted fluorescence microscopy images (a), photograph (b), average diameter (c) and EI% (d) of Pickering emulsions at different NaCl concentrations (0.20 wt%, 0.40 wt%, 0.60 wt%, 0.80 wt%, 1.0 wt% and 2.0 wt%; $\omega_{(TOCNFs)}$ = 0.9 wt%, pH = 4, 450 W).

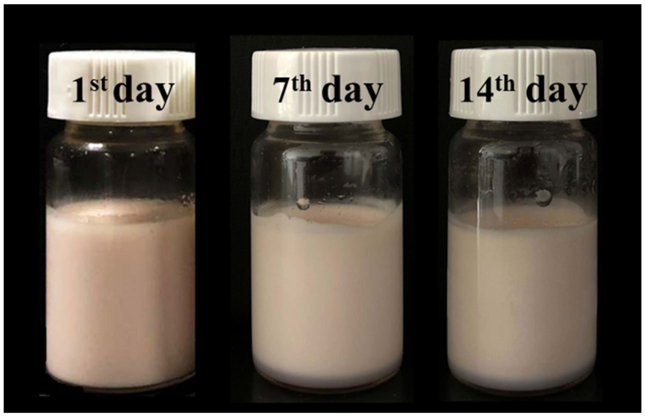

Figure S3. Photographs of AXT-loaded Pickering emulsions stored for 1 day, 7 days and 14 days.
